# Biosafety level-2 laboratory diagnosis of Zaire Ebola virus disease imported from Liberia to Nigeria

**DOI:** 10.4102/ajlm.v5i1.468

**Published:** 2016-10-17

**Authors:** Olumuyiwa B. Salu, Ayorinde B. James, Bamidele O. Oke, Mercy R. Orenolu, Roosevelt A. Anyanwu, Maryam A. Abdullah, Christian Happi, Jide Idris, Ismail A. Abdus-Salam, Abdul-Salam Nasidi, Folashade T. Ogunsola, Oyewale Tomori, Sunday A. Omilabu

**Affiliations:** 1 Department of Medical Microbiology and Parasitology, College of Medicine, University of Lagos, Lagos, Nigeria; 2 Department of Biochemistry, College of Medicine, University of Lagos, Lagos, Nigeria; 3 Central Research Laboratory, College of Medicine, University of Lagos, Lagos, Nigeria; 4 African Center of Excellence for Genomics of Infectious Diseases, Redeemers University, Ede, Osun State, Nigeria; 5 Department of Biological Sciences, College of Natural Sciences, Redeemers University, Ede, Osun State, Nigeria; 6 Honourable Commissioner for Health, Lagos State Ministry of Health, Alausa, Ikeja, Lagos, Nigeria; 7 Epidemiology Unit, Directorate of Disease Control, Lagos State Ministry of Health, Alausa, Ikeja, Lagos, Nigeria; 8 Nigeria Center for Disease Control, Federal Ministry of Health, Abuja, Nigeria; 9 Nigerian Academy of Science, Lagos, Nigeria

## Abstract

**Introduction:**

Global travel is an efficient route of transmission for highly infectious pathogens and increases the chances of such pathogens moving from high disease-endemic areas to new regions. We describe the rapid and safe identification of the first imported case of Ebola virus disease in a traveler to Lagos, Nigeria, using conventional reverse transcription polymerase chain reaction (RT-PCR) in a biosafety level (BSL)-2 facility.

**Case presentation:**

On 20 July 2014, a traveler arrived from Liberia at Lagos International Airport and was admitted to a private hospital in Lagos, with clinical suspicion of Ebola virus disease.

**Methodology and Outcome:**

Blood and urine specimens were collected, transported to the Virology Unit Laboratory at the College of Medicine, University of Lagos, and processed under stringent biosafety conditions for viral RNA extraction. RT-PCR was set-up to query the Ebola, Lassa and Dengue fever viruses. Amplicons for pan-filoviruses were detected as 300 bp bands on a 1.5% agarose gel image; there were no detectable bands for Lassa and Dengue viral RNA. Nucleotide BLAST and phylogenetic analysis of sequence data of the RNA-dependent RNA polymerase (L) gene confirmed the sequence to be *Zaire ebolavirus* (EBOV/Hsap/NGA/2014/LIB-NIG 01072014; Genbank: KM251803.1).

**Conclusion:**

Our BSL-2 facility in Lagos, Nigeria, was able to safely detect Ebola virus disease using molecular techniques, supporting the reliability of molecular detection of highly infectious viral pathogens under stringent safety guidelines in BSL-2 laboratories. This is a significant lesson for the many under-facilitated laboratories in resource-limited settings, as is predominantly found in sub-Saharan Africa.

## Introduction

Ebola virus is the causative agent of Ebola virus disease (EVD), previously known as Ebola haemorrhagic fever.^1^ The genus *Ebolavirus* belongs to the *Filoviridae* family, alongside *Marburgvirus* and the newly-identified genus *Cuevavirus*.^1^ The *Ebolavirus* genus consists of five different species, including *Zaire ebolavirus, Sudan ebolavirus, Bundibugyo ebolavirus, Tai Forest ebolavirus* and *Reston ebolavirus.^2^* The first three have been associated with major disease outbreaks in West Africa, whereas *Tai Forest ebolavirus* has one reported case of human infection in an individual who had contact with an infected chimpanzee from the Tai forest in Ivory Coast and *Reston ebolavirus* has been reported in the Asia-Pacific region, but with no recorded cases of human infection.^2,3,4^ Members of the family *Filoviridae* in the order of *Mononegavirales* are all non-segmented negative-stranded RNA viruses. Their genomic RNA contains filoviral genes that are organised in the following linear order: 3’-(leader)-NP-VP35-VP40-GP-VP30-VP24-L-(trailer)-5’.^5,6^

On 17 July 2014, the Ministries of Health in Liberia, Guinea and Sierra Leone, in collaboration with the World Health Organization, announced a cumulative total of 1048 suspected and 745 laboratory-confirmed cases of EVD, with 632 (60.3%) deaths^7^. Blood and urine specimens were sent to the Virology Unit Laboratory of the Central Research laboratory, College of Medicine (CMUL)-University of Lagos and Lagos University Teaching Hospital (LUTH). This report presents the laboratory methods used, including conventional reverse transcription polymerase chain reaction (PCR), to diagnose the first imported case of EVD in Nigeria in a biosafety level (BSL)-2 facility.

## Case presentation

**FIGURE 1:**
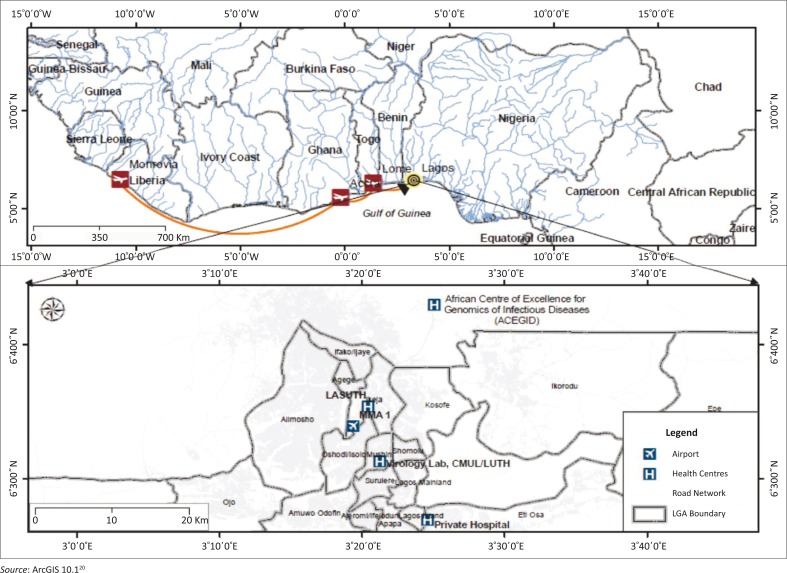
Travel route of index case with Ebola virus disease from Liberia to Lagos, Nigeria. Lower inset shows the closest health care facility to the airport (LASUTH), the private health care facility where the index case was admitted (Private Hospital), the Laboratory where the diagnosis was made (Virology Lab, CMUL/LUTH) and the Genomics Centre where the corroborative diagnosis and nucleotide sequencing was done (ACEGID). Map was plotted with GPS coordinates using Arc GIS 10.1.

A 40-year-old Liberian man, who had been living in Elwa, Monrovia, travelled by air through Accra (Ghana) and Lomé (Togo) to Lagos, Nigeria. Upon arrival at Lagos International Airport on 20 July 2014, he collapsed and was taken to a private hospital in the Obalende area of Lagos, Nigeria (Figure 1), where he was admitted. He presented with high fever, lymphadenopathy and sore throat and was treated for malaria and typhoid fever until he later developed diarrhoea, vomiting and microscopic haematuria. The culmination of the clinical presentation, the patient’s non-response to treatment for malaria and typhoid, as well as his epidemiological link to Liberia, raised the suspicion for EVD. On 22 July 2014, blood samples in EDTA tubes and urine samples were collected and sent for viral investigations to the CMUL-LUTH Virology Unit Laboratory. Aliquots of the specimens were sent in parallel to the African Center of Excellence for Genomics of Infectious Diseases (ACEGID) at Redeemers University in Ogun State, Nigeria.

### Ethical considerations

No ethics approval was required for the diagnosis of an infection of great public health importance and which was part of the normal course of management of any infected patient.

## Management and Outcomes

### Specimen handling and processing

Due to the fact that no BSL-3 or BSL-4 facilities were available to handle the patient’s specimens, all samples received were handled in a BSL-2 facility with extreme care and in line with US Centers for Disease Control and Prevention (CDC) safety guidelines.^8^ Personal protective equipment (PPE) was worn in accordance with the ‘donning and doffing of PPE’ protocol set out by the CDC.^8^ Sample carrier boxes were sprayed with 10% hypochlorite solution on a disposable laboratory absorbent mat in a biosafety class IIA cabinet. Specimen containers were removed from the carrier box and wrapped immediately with a laboratory paper towel soaked in 10% hypochlorite in order to disinfect any possible spillage around the specimen container. An identical process was used for removal of samples from the container box. Outer gloves were sprayed with 10% hypochlorite solution every time hands were withdrawn from the safety cabinet and then disposed of in double-jacketed bio-hazard waste bags if they were soiled during sample handling or at the end of the procedure. Aliquots of specimens used for viral assays were immediately inactivated in a guanidinium-thiocyanate lysis buffer for viral nucleic acid extraction (Qiagen, Germantown, Maryland, United States).

### Viral RNA isolation and RT-PCR

For viral RNA extraction, neat (undiluted), 1:10 and 1:100 dilutions in sterile phosphate-buffered saline specimens of both blood and urine were processed using a Qiagen RNA extraction kit (Qiagen, Germantown, Maryland, United States). Segments of the L-gene of the Ebola virus, the S-gene of the Lassa virus and the 3’ non-coding region of the Dengue fever virus were amplified in singleplex PCRs using the primers listed in Table 1. Separate master mixes for each queried virus were prepared and cycled as described in the AmbionAgPath-ID One-Step RT-PCR kit (Applied Biosystems, Foster City, California, United States) protocol. Subsequently, PCR amplicons were subjected to 1.5% agarose gel electrophoresis with 1X SYBR^®^ Safe DNA gel staining dye (Invitrogen, Carlsbad, California, United States) at 120V for 30 minutes and images were taken with a BioDocAnalyze 2.0 (Biometra, Goettingen, Germany).

**TABLE 1:** Primers used in the laboratory investigation of viral haemorrhagic diseases.

Virus	Primer	Sequence	Amplicon size (bp)
Lassa fever virus	36E2 LVS-339-rev	5’-GTT CTT TGT GCA GGA MAG GGG CAT KGT CAT-3’ 5’-ACC GGG GAT CCTAGG CAT TT-3’	~300
Dengue fever virus	DenS DenAs- REV DenAs+ -REV	5’-GGA TAG ACC AGA GAT CCT GCT GT-3’ 5’-CAT TCC ATT TTC TGG CGT TC-3’ 5’-CAG CAT CAT TCC AGG CAC AG-3’	97
Filovirus strains	FiloA2.2 FiloA2.3 FiloA2.4 FiloB-REV FiloB_RAVN_-REV	5’-AAG CCT TTC CTA GCA ACA TGA TGG T-3’ 5’-AAG CAT TCC CTA GCA ACA TGA TGG T-3’ 5’-AAGCATTTCCTAGCAATATGATGGT-3’Ç 5’-ATG TGG TGG GTT ATA ATA ATC ACT GAC ATG-3’ 5’-TGA GGA GGG CTA TAA AAG TCA CTG ACA TG-3’	298

*Source*: Drosten et al.^15^

### EBOV L-Gene sequencing and phylogenetic analysis

**FIGURE 2:**
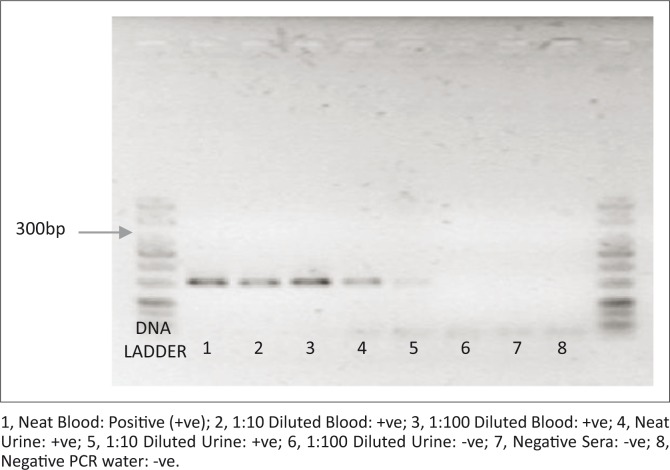
Agarose gel image showing PCR amplicon bands of the specimens. The ~300 bp band is the target band size for Filoviruses.

PCR amplicons on the agarose gel were purified using an agarose gel extraction kit (Jena Bioscience, Jena, Germany). Purified non-infectious PCR products, packaged and transported using triple-level packaging, were sequenced using the Filo A2.3 primer on the Sanger dideoxy sequencing technology platform with an Applied Biosystems 3130xl Genetic Analyser at Jena Bioscience in Jena, Germany. A sequence trace file was used for BLAST analysis in GenBank.^9^ The RNA-dependent RNA polymerase (L) gene, partial Coding sequence (CDS) for EBOV/Hsap/NGA/2014/LIB-NIG 01072014 was deposited in the National Center for Biotechnology Information (NCBI) with the accession number KM251803.1. A set of 30 different Filovirus genomes were selected from NCBI’s GenBank. The FASTA format of the L-gene region of all the selected genomes was downloaded and aligned with the KM251803.1 sequence using the MUSCLE tool of MEGA-6 software^9,10^ (Figure 2). The Tamura 3 parameter (T92) was found to be the best maximum likelihood model to infer the phylogenetic tree. A discrete Gamma distribution was used to model evolutionary rate differences among sites (*+G*, parameter = 0.8048) and the rate variation model allowed for some sites to be evolutionarily invariable ([*+I*], 36.3524% sites).^11^ The reliability of each node on the phylogenetic tree was tested by bootstrapping with 1000 replicates.

### RT-PCR detection of queried haemorrhagic viruses

Investigations carried out on viral RNA purified from the blood and urine specimens of the patient revealed the presence of the expected amplicon size (300 bp) for Filoviruses (Figure 2). However, no signals were observed for Lassa or Dengue fever viruses. The filovirus amplicons were still detected in blood specimens with logscale dilutions of 1 (1:10) and 2 (1:100), whereas in urine samples the signal was no longer detectable at a log 3 dilution.

### Phylogenetic analysis of RNA-dependent RNA-polymerase L-Gene partial CDS

**FIGURE 3:**
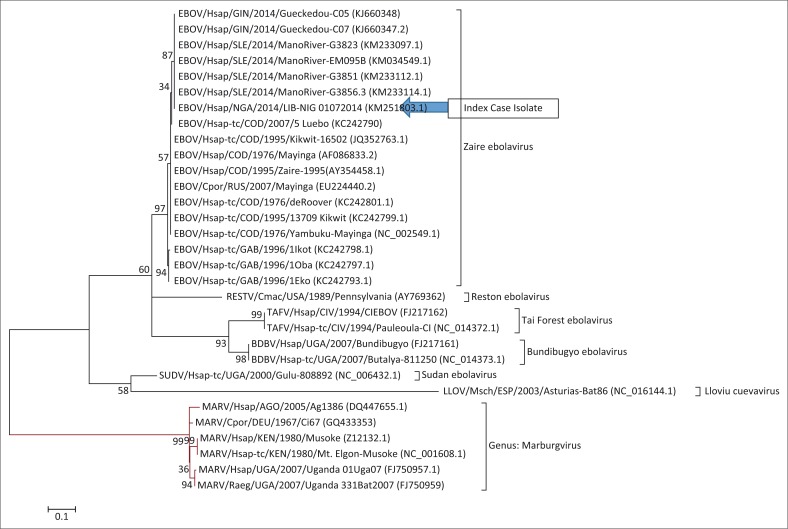
Molecular phylogenetic analysis of the L-gene segment of EBOV/Hsap/NGA/2014/LIB-NIG 01072014 in comparison with selected filoviruses sequences by the maximum likelihood method. Sequences are labelled using the ICTV consensus nomenclature for variants of the *Filoviridae* family with their corresponding Genebank Accession numbers in parenthesis.

Phylogenetic analysis of the L-gene partial CDS indicated that the virus evolved from the Guinea and Mano River isolates of the 2014 outbreak with a bootstraping value of 87% (Figure 3). Other isolates represented on the phylogenetic tree confirmed the evolutionary relationships between the different filoviruses.

## Discussion

By using molecular techniques and following CDC safety guidelines, our BSL-2 laboratory was able to safely identify the Ebola virus in blood and urine samples. The clinical symptoms of the index case were initially linked to malaria and typhoid fever, until the patient began to haemorrhage, which led to a differential diagnosis of Lassa, Ebola or Dengue fever. The time lapse between hospital admission, malaria/typhoid treatment and the clinical suspicion of EVD was two days and led to the exposure of 72 persons at the airport and the hospital.^12^ This relatively quick diagnosis of the disease once the specimens reached the virology laboratories at CMUL-LUTH and ACEGID was significant in limiting the extent of the outbreak, unlike the situation in Guinea where the infection spread undetected for months, leading to the catastrophic epidemic in the West African sub-region.^13^

Partial genomic sequence analysis data identified *Zaire ebolavirus* as the aetiological agent of the disease (GenBank Accession: KM251803.1). Phylogenetic analysis of the RNA-dependent RNA polymerase (L) gene, partial CDS showed a clustering (87%) with the *Zaire ebolavirus* sequences originating from Gueckedou and Mano River in 2014. This finding corroborates the clinical suspicion and the travel history of the index case to Nigeria. According to Kuhn et al., the Ebola virus responsible for the West African outbreak is the Makona variant.^14^ The name ‘Makona’ originated from the Makona River which is central to the epicentre of the 2014 outbreak.^14^

Many African countries, governments and research institutions are inadequately equipped or trained in diagnostics, active surveillance and reporting of highly infectious diseases.^3^ However, a number of centres such as ours are now being equipped to train scientists and scale up surveillance activities in the event of another outbreak. Although we had no previous experience in diagnosing Ebola virus, our involvement in the diagnosis of Lassa virus, which is endemic in Nigeria, led to the development of infrastructure for its molecular diagnosis under stringent biosafety procedures. Because of collaborations with the Bernard Notch Institute for Tropical Infections in Germany, our laboratory had acquired sets of primers for diagnosis and surveillance of Ebola, Lassa, Crimean-Congo, Rift Valley, Dengue and Yellow fever viruses over the past 14 years.^15^ During that time, our laboratory grew to serve various hospital diagnostics and outbreak investigations in Nigeria with funds provided by research granting bodies such as Laboratoire National de Santé Institute, CRP-Santé, Luxembourg, and the German Research Council, Germany. Thus, in collaboration with the Bernard Notch Institute for Tropical Infections, we have been in the forefront of viral haemorrhagic fever investigations and surveillance in Nigeria.

### Limitations

Molecular detection using PCR is the most sensitive method for viral diagnostics. The use of chaotropic lysis buffer for the isolation of viral nucleic acids from the highly contagious specimens was a key step, because it rendered specimens non-infectious and thus safe for processing in a BSL-2 laboratory. However, despite our success, it should be noted that manipulations of highly pathogenic viruses such as Ebola cannot be attempted in a BSL-2 laboratory. Manipulations, such as culturing live Ebola viruses, must be restricted to BSL-4 and BSL-3-enhanced laboratories, because of the higher risk of contracting Ebola virus when incidents occur.^16,17,18,19^ Thus, in our case the sequencing of the detected Ebola virus was done at Jena Bioscience in Germany.

### Conclusion

One of the major lessons learnt from this outbreak is that that there is an urgent need to build capability for rapid detection of and response to disease outbreaks in resource-limited countries, especially in West Africa where the health systems are very weak. This should serve as a wake-up call, not only for African governments but also to the world, that investment in laboratory infrastructure and improvements in laboratory capabilities, as well as building capacity for disease surveillance, infection control and biosafety, is critical so that these countries do not constitute the weak links in the ongoing fight against infectious diseases.
